# Dihydrocapsaicin Inhibits Cell Proliferation and Metastasis in Melanoma *via* Down-regulating β-Catenin Pathway

**DOI:** 10.3389/fonc.2021.648052

**Published:** 2021-03-23

**Authors:** Shaomin Shi, Chongyang Li, Yanli Zhang, Chaowei Deng, Wei Liu, Juan Du, Qian Li, Yacong Ji, Leiyang Guo, Lichao Liu, Huanrong Hu, Yaling Liu, Hongjuan Cui

**Affiliations:** ^1^ Department of Dermatology, Third Hospital of Hebei Medical University, Shijiazhuang, China; ^2^ State Key Laboratory of Silkworm Genome Biology, Southwest University, Chongqing, China; ^3^ Cancer Center, Medical Research Institute, Southwest University, Chongqing, China; ^4^ Department of Dermatology, Fifth Hospital of Shijiazhuang, Shijiazhuang, China

**Keywords:** dihydrocapsaicin, melanoma, cell proliferation, metastasis, β-catenin, ubiquitination

## Abstract

Dihydrocapsaicin (DHC) is one of the main components of capsaicinoids in Capsicum. It has been reported that DHC exerts anti-cancer effects on diverse malignant tumors, such as colorectal cancer, breast cancer, and glioma. However, studies focused on the effect of DHC upon melanoma have rarely been done. In the present study, melanoma A375 and MV3 cell lines were treated with DHC and the cell proliferation, migration, and invasion were significantly suppressed. Furthermore, DHC effectively inhibited xenograft tumor growth and pulmonary metastasis of melanoma cells in NOD/SCID mice model. It was identified that β-catenin, which plays significant roles in cell proliferation and epithelial-mesenchymal transition, was down-regulated after DHC treatment. In addition, cyclin D1, c-Myc, MMP2, and MMP7, which are critical in diverse cellular process regulation as downstream proteins of β-catenin, were all decreased. Mechanistically, DHC accelerates ubiquitination of β-catenin and up-regulates the beta-transducin repeat containing E3 ubiquitin protein ligase (BTRC) in melanoma cells. The DHC induced suppression of cell proliferation, migration, and invasion were partly rescued by exogenous β-catenin overexpression, both *in vitro* and *in vivo*. Taken together, DHC may serve as a candidate natural compound for human melanoma treatment through β-catenin pathway.

## Introduction

Natural ingredients extracted from plants are commonly used in medical research for their wide distribution and easy access. In recent years, an increasing number of natural anti-cancer compounds have been discovered (1). Compared with synthetic drugs, plant-derived agents are usually better tolerated out of less toxicity to human cells (2). Capsaicin (Cap) and DHC are the main active components in Capsicum that bring numerous health benefits (3). The apoptosis induction of Cap has been extensively studied in multiple cancer cells, such as glioma, osteosarcoma, hepatocarcinoma, renal carcinoma, and melanoma (4–8). DHC also shows the effect of apoptosis induction in human glioma cells through reactive oxygen species (ROS) and Ca2^+^-mediated mitochondrial pathway (9). Moreover, DHC exhibited more potently proliferation inhibition activity on HCT116 colon cancer cells and MCF-7 breast cancer cells than Cap (10). Although Cap has been reported to promote apoptosis and attenuates migration in melanoma cells (8, 11), the anti-cancer effect of DHC in melanoma remains unclear.

Cutaneous melanoma is a highly malignant tumor originated from melanocytes in the basal layer of the epidermis. Although melanoma accounts merely 5% of all cutaneous malignancies, it occupies the 90% of skin cancer-related deaths (12, 13). The incidence of melanoma has been continuously increasing in recent decades throughout the world, especially in Caucasian population (12, 13). In China, the age-standardized incidence rates of melanoma had doubled from 1990 to 2017 (14). Unfortunately, a large proportion of melanoma patients were diagnosed with locally advanced stage and metastatic disease (15).

For early stage melanoma, surgery remains the mainstay of treatment with a high cure rate (16). However, prognosis of advanced melanoma is dismal because of its resistance to conventional therapies, including chemotherapy and radiotherapy (17). Nevertheless, with the development of targeted therapies and immunotherapies, major breakthroughs have been made in the treatment of patients with advanced melanoma, which substantially improved the overall survival (18, 19). Combination of anti-CTLA-4 and anti-PD-1 was shown to significantly enhance the efficacy in metastatic melanoma patients (20). BRAF inhibitors, alone and in combination with MEK inhibitors, significantly improve the progression-free survival among advanced melanoma patients (21, 22). Depressingly, there is still a substantial proportion of patients that do not respond to these treatments or develop resistance, and the severe side effects of immunotherapy are fatal for patients (19, 23). Therefore, novel effective drugs with low toxicity for the treatment of melanoma still need to be explored.

β-catenin is a multitasking and evolutionary conserved molecule that exerts a crucial role in a multitude of physiological and pathological processes (24). Abnormal regulation of β-catenin has been implicated in a variety of human disease, including diverse cancers (25, 26). In previous studies, β-catenin was shown to promote cell proliferation, invasiveness, and chemoresistance in melanoma (27–29). Therefore, β-catenin has become one of the potential targets in anti-melanoma research. For instance, some compounds have been shown to inhibit melanoma by regulating the Wnt/β-catenin signaling pathway (28, 30).

In the present study, we demonstrated that DHC inhibited the proliferation and metastasis of human melanoma cells both *in vitro* and *in vivo* by a β-catenin dependent way. Further research revealed that DHC could up-regulate the expression of BTRC and accelerate the ubiquitination of β-catenin. Therefore, DHC may serve as a candidate natural compound for human melanoma treatment through β-catenin pathway.

## Materials and Methods

### Cell Culture and DHC Treatment

Human A375 and MV3 melanoma cells, PIG1 melanocytes, HaCaT keratinocytes, and 293-FT human embryonic renal cell lines were obtained from the American Type Culture Collection (ATCC, Rockville, MD, USA). DHC with more than 99% purity was purchased from Must Bio-Technology (Chengdu, China). A375, PIG1, and HaCaT cells were cultured in Dulbecco’s Modified Eagle’s Medium (DMEM, Gibco, New York, NY, USA), MV3 cells were cultured in Roswell Park Memorial Institute-1640 (RPMI-1640, Gibco), both supplemented with 10% fetal bovine serum (FBS, Gibco) and 1% penicillin-streptomycin (Gibco). The culture medium used for 293FT cells was DMEM supplemented with 10% FBS, 2% glutamine (Invitrogen, Carlsbad, CA, USA), 1% non-essential amino acids (Invitrogen), 1% sodium pyruvate (Invitrogen), and 1% G418 (Invitrogen). All cells were tested free of mycoplasma and cultured in a humidified incubator with 5% CO_2_ at 37°C. A375 and MV3 cells were treated with DHC at indicated concentrations for different time, equivalent volumes of dimethyl sulfoxide (DMSO, Sigma-Aldrich, Shanghai, China) was used as control.

### MTT Assays

Cells in amount of 1 × 10^3^/well were seeded in 96-well plates and incubated for overnight, then further cultured with multiple doses of DHC for 48 h. Afterwards, 20 μl MTT (5 mg/ml) was added to each well for an additional 2 h of incubation and the generated formazan were resolved with 200 μl DMSO. The absorbance was measured at 560 nm with a microplate reader (Thermo Fisher, Waltham, MA, USA). By virtue of the GraphPad Prism 6.0, IC50 value of DHC for A375 and MV3 cells, as well as PIG1 and HaCaT cells, were calculated. In the same way, cell viability of A375, MV3, PIG1, and HaCaT cells after DHC treatment was detected.

### Edu Staining Assays

A total of 1 × 10^4^ cells were added to each well of 24-well plates and incubated for 8 h, then cells were exposed to 100 μM DHC or isometric DMSO for 48 h. EdU staining was performed according to the instructions of BeyoClick™ EdU Cell Proliferation Kit (Beyotime, Shanghai, China). The number of EdU-positive cells was observed and recorded under a fluorescence microscope (Olympus, Tokyo, Japan).

### Flow Cytometry Assays

Cells were incubated in six-well plates containing 100 μM DHC for 48 h, DMSO was used as control. After incubating, the cells were collected and fixed in 75% ethanol at 4°C for 24 h. After being washed with PBS, the cells were stained with FxCycle™ PI/RNase Staining Solution (Invitrogen, Carlsbad, CA, USA) according to manufacturer’s instructions. Cell cycle was identified by BD Accuri C6 flow cytometer (BD, San Jose, CA, USA) and the FlowJo Software version 7.6.1 (FlowJo LLC, Ashland, OR, USA) was used for further analyses.

### Wound-Healing Assays

Cells were cultured in six-well plates and grown to 80–90% confluence. Then, a scratch line was etched on the monolayer of the cells with a 200 µl pipette tip. Serum-free medium with 100 μM DHC was used for subsequent culturing, DMSO was used as control. Width of the wounds were examined and imaged at 0, 24, 48, and 72 h with an inverted microscope (Nikon, Tokyo, Japan). The wound closure proportion was calculated as: (wound width at 0h − wound width at 24, 48, and 72 h)/wound width at 0 h × 100%.

### Migration and Invasion Transwell Assays

The 8-μm transwell chambers (Corning, Beijing, China) were used for migration assay, while for invasion assay, the chambers were pre-loaded with 50 µl of Matrigel (BD). Firstly, 200 µl serum-free medium mixed with 1 × 10^5^ cells and 100 μM DHC was added into the upper chambers, DMSO was used as control. Then the upper chambers were placed into the lower compartments which loaded with 500 µl medium containing 10% FBS. After 16 or 24 h incubation for migration and invasion assays respectively, the non-migrating and non-invading cells remained on the upper chambers were carefully wiped off. Afterwards, the chambers were dyed with crystal violet staining solution (Beyotime) and the cells being stained were quantified under an inverted microscope (Nikon).

### qRT-PCR

Total RNA was extracted by TRIzol Reagent (Invitrogen). Then, 2 μg of RNA was reverse transcribed into complementary DNA using M-MLV reverse transcriptase (Promega, WI, USA) for each sample. GoTaq^®^ qPCR Master Mix (Promega) was used for qRT-PCR. mRNA expression was calculated based on Ct values and was normalized by the values of GAPDH. See details in previous paper (31). All quantitative primers are listed in **Table 1**.

**Table 1:** Primers used in qRT‐PCR.

Genes	Primers (5’-3’)
cyclin D1	F: 5’-GCTGCGAAGTGGAAACCATC-3’ R: 5’-CCTCCTTCTGCACACATTTGAA-3’
c-Myc	F: 5’-GGCTCCTGGCAAAAGGTCA-3’ R: 5’-CTGCGTAGTTGTGCTGATGT-3’
cyclin A2	F: 5’-GGATGGTAGTTTTGAGTCACCAC-3’ R: 5’-CACGAGGATAGCTCTCATACTGT-3’
β-catenin	F: 5’-AAAGCGGCTGTTAGTCACTGG-3’ R: 5’-CGAGTCATTGCATACTGTCCAT-3’
MMP2	F: 5’-CCCACTGCGGTTTTCTCGAAT-3’ R: 5’-CAAAGGGGTATCCATCGCCAT-3’
MMP7	F: 5’-GAGTGAGCTACAGTGGGAACA-3’ R: 5’-CTATGACGCGGGAGTTTAACAT-3’
GAPDH	F: 5’-GGAGCGAGATCCCTCCAAAAT-3’ R: 5’-GGCTGTTGTCATACTTCTCATGG-3’

### Western Blotting

Cells were lysed using ice-cold RIPA lysis buffer (Beyotime) and the protein concentrations were quantified. Western blotting assay was performed as previously described (32). The PVDF membranes were incubated with rabbit anti-cyclin D1 (1:1,000; Proteintech, Wuhan, China), c-Myc (1:1,000; Cell Signaling, Boston, MA, USA), cyclin A2 (1:1,000; Cell Signaling), β-catenin (1:1,000; Cell Signaling), MMP2 (1:1,000; Proteintech), MMP7 (1:1,000; Cell Signaling), BTRC (1:1,000; Cell Signaling), FBXW7 (1:1,000; Proteintech), and α-tubulin (1:2,000; Proteintech) primary antibodies at 4°C for overnight. After conjugated with HRP goat anti-rabbit IgG antibody (1:5,000; Abcam, Cambridge, MA, USA), immunoreactive bands were visualized by the Super ECL prime (US Everbright Inc., Suzhou, China) under a western blotting detection instrument (Clinx Science Instruments, Shanghai, China).

### Soft Agar Colony Formation Assays

Briefly, 1.5 ml of complete medium containing 0.6% agarose (Sigma-Aldrich) and 100 μM DHC was added to each well of the six-well plates as base agar. After the base agar solidified, 1 ml of top agar constructed with complete medium, 0.3% agarose, 1 × 10^3^ cells, and 100 μM DHC was overlaid onto its surface. The plates were incubated in a 37°C, 5% CO_2_ incubator for 3 weeks, and the colonies were counted with a microscope after MTT staining.

### Vector Construction and Infection

For the over-expression of β-catenin in melanoma cells, full-length human β-catenin cDNA was cloned into pCDH-CMV-MCS-EF1-GFP+Puro lentiviral vector (Unibio, Chongqing, China). Lentiviral particles containing β-catenin were packaged by Lipofectamine™ 2000 transfection reagent (Thermo Fisher) according to the manufacturer’s instruction, empty vector was used as control. Subsequently, A375 and MV3 cells were infected with concentrated lentivirus and were selected with 4 μg/ml puromycin (Sigma-Aldrich) to generate stable transfected cell lines.

### *In Vivo* Tumor Xenograft Assays

Four-week-old female NOD/SCID mice were purchased from HFK Bioscience Co., Ltd (Beijing, China) and kept in an institutional facility under facility specific pathogen-free (SPF) conditions. Animal experiments were approved by the Committee for Animal Protection and Utilization of Southwest University. Three types of A375 cells (parental, empty vector overexpressed, and β-catenin overexpressed) in amount of 1 × 10^6^ were subcutaneously injected into the right flank region of eight mice respectively. Then we have three groups of mice. When tumors were palpable 7 days later, each group of mice was randomized into two parts (four in each part) and intraperitoneally injected with 20 mg/kg DHC or isometric DMSO once a day for 28 days. Tumor width and length were measured with a caliper every 4 days and the tumor volumes were calculated as follows: volume = tumor length × width^2^ × π/6. At the end, mice were euthanized, tumor from each mouse was weighed.

### Immunohistochemistry (IHC) Staining

The xenograft tumors were immobilized, dehydrated, and paraffin embedded, 5-μm thick sections were stained with β-catenin (1:400; Cell Signaling) and Ki67 (1:200; Abcam) for IHC analysis. Detailed steps were performed with reference to a previously study (33).

### *In Vivo* Metastasis Assays

Three types of A375 cells (parental, empty vector overexpressed and β-catenin overexpressed) in amount of 5 × 10^5^ were injected into the tail veins of eight NOD/SCID mice respectively once a day for 3 days. Then we have three groups of mice. Each group of mice was randomly divided into two parts (four in each part) on the fourth day, and intraperitoneally injected with 20 mg/kg DHC or isometric DMSO respectively once a day for 45 days. After that, the mice were euthanized and lungs were collected. H&E staining was performed to observe the pulmonary metastasis tumors.

### Protein Ubiquitination and Turnover Assays

For ubiquitination assays, HA-ubiquitin plasmids were transfected into 293FT cells. After pretreat with 100 μM DHC or DMSO for 16 h, 20 µg/ml proteasome inhibitor MG132 (Sigma-Aldrich) was added to the cells and incubated for another 8 h. Then the cells were collected and lysed and equal amount of protein from each sample was incubated with β-catenin antibody (1:100; Cell Signaling) overnight at 4°C. Protein A/G Magnetic Beads (Beyotime) were added to adsorb the immunoprecipitants and suspended with 1× loading buffer for western blotting. Ubiquitin antibody (1:1,000; Abcam) was used to check the interaction between β-catenin and ubiquitin. For the turnover assay, DHC and DMSO pretreated A375 cells were incubated with 50 µg/ml cycloheximide (CHX, Sigma-Aldrich) for indicated time in the presence or absence of DHC. Then, cells were collected, lysed and the protein levels of β-catenin were assessed by western blotting.

### Molecular Docking

The molecular structure of DHC in mol format file (ID: 97096) was downloaded from ChemSpider (http://www.chemspider.com/). Crystal structures of β-catenin (ID: 2Z6H) and BTRC (ID: 2p64) were obtained from PROTIEN DATA BANK (https://www.rcsb.org/) and saved as PDB format. Molecular docking analysis of DHC with β-catenin and BTRC was performed by iGEMDOCK tool. Bond energy score was identified to judge the degree of intermolecular bonding.

### Statistical Analysis

All experiments were performed at least three independent repetitions. Data are expressed as mean ± SD. Statistical analyses were performed using GraphPad Prism 6.0. Statistical significance between two groups was analyzed by two-tailed unpaired Student’s t-test. One-way ANOVA was used to assess the mean difference among three or more groups, further multiple comparisons were done with Tukey’s test. The value of *P* < 0.05 was considered as statistically significant and denoted as *P < 0.05, **P < 0.01, and ***P < 0.001.

## Results

### DHC Inhibits Melanoma Cells Proliferation

**Figure 1:**
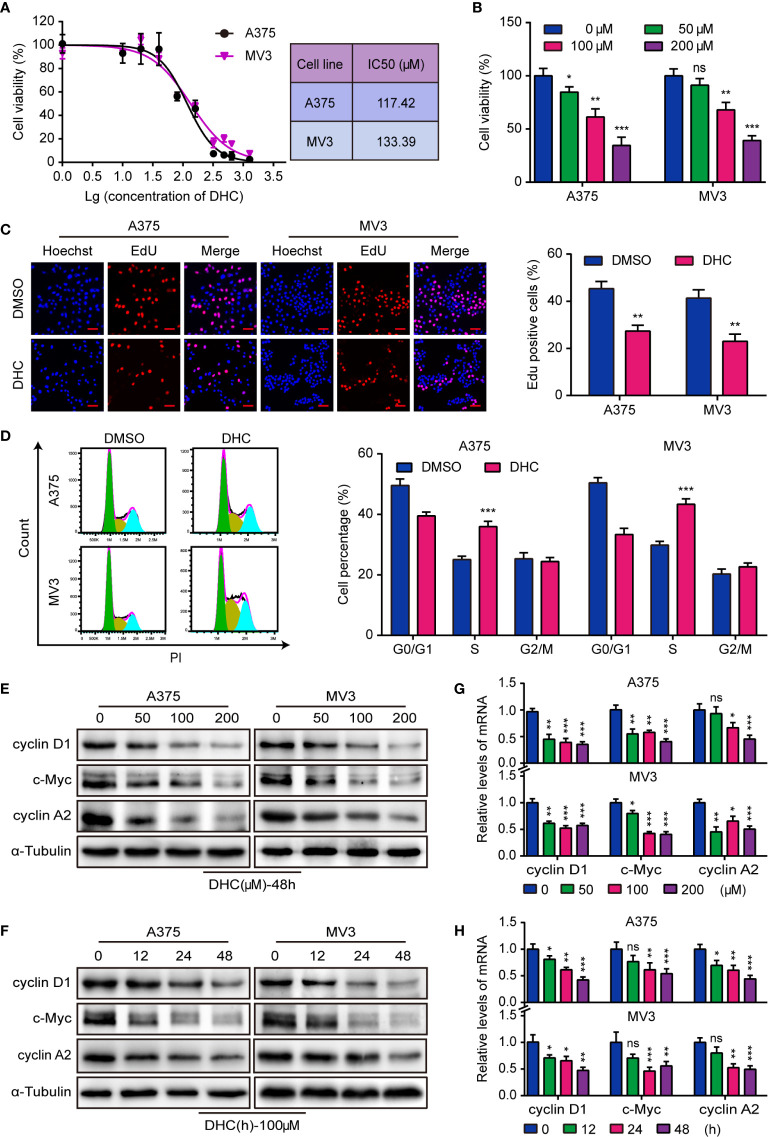
DHC shows anti-proliferation activity in melanoma cells. **(A)** The IC50 of DHC for A375 and MV3 cells was measured. **(B)** Cell viabilities of A375 and MV3 cells treated with DHC (0, 50, 100, and 200 μM) for 48 h. **(C)** EdU staining of A375 and MV3 cells treated with 100 μM DHC for 48 h. Scale bar, 50 μm. **(D)** Cell cycle analysis of A375 and MV3 cells treated with 100 μM DHC for 48 h. **(E, G)** The protein and mRNA levels of cyclin D1, c-Myc, and cyclin A2 in A375 and MV3 cells treated with 0, 50, 100, and 200 μM DHC for 48 h. **(F, H)** The protein and mRNA levels of cyclin D1, c-Myc, and cyclin A2 in A375 and MV3 cells treated with 100 μM DHC for 0, 12, 24, and 48 h. *P < 0.05; **P < 0.01; ***P < 0.001; ns, not significant.

The IC50 of DHC for two melanoma cell lines, A375 and MV3, were 117.42 and 133.39 μM respectively (**Figure 1A**). Meanwhile, the IC50 of DHC for PIG1 and HaCaT cells were 192.30 and 238.50 μM, which were higher than that of melanoma cells (**Figure S1A**). It is worth mentioning that DHC in 100 μM has no significant influences on the viability of PIG1 and HaCaT cells (**Figure S1B**). According to the IC50, A375 and MV3 cells were treated with 0, 50, 100, and 200 μM of DHC for 48 h. As data shown, the viability of melanoma cells was evidently inhibited following DHC treatment in a dose-dependent manner (**Figure 1B**). Furthermore, the percentage of EdU positive cells was notably decreased after DHC treatment (**Figure 1C**). According to the results of cell cycle analysis, the proportions of S phase in both cell lines were significantly increased after treated with DHC (**Figure 1D**). In addition, the expression of cyclin D1, c-Myc, and cyclin A2 proteins, which are related to cell proliferation, was decreased in a dose- and time-dependent manner in DHC treated cells (**Figures 1E, F**), while the mRNA expression levels of those factors were decreased in the same pattern (**Figures 1G, H**). These findings demonstrated that DHC inhibits melanoma cells proliferation and blocks the cell cycle at S phase.

### DHC Inhibits Melanoma Cells Migration and Invasion

**Figure 2:**
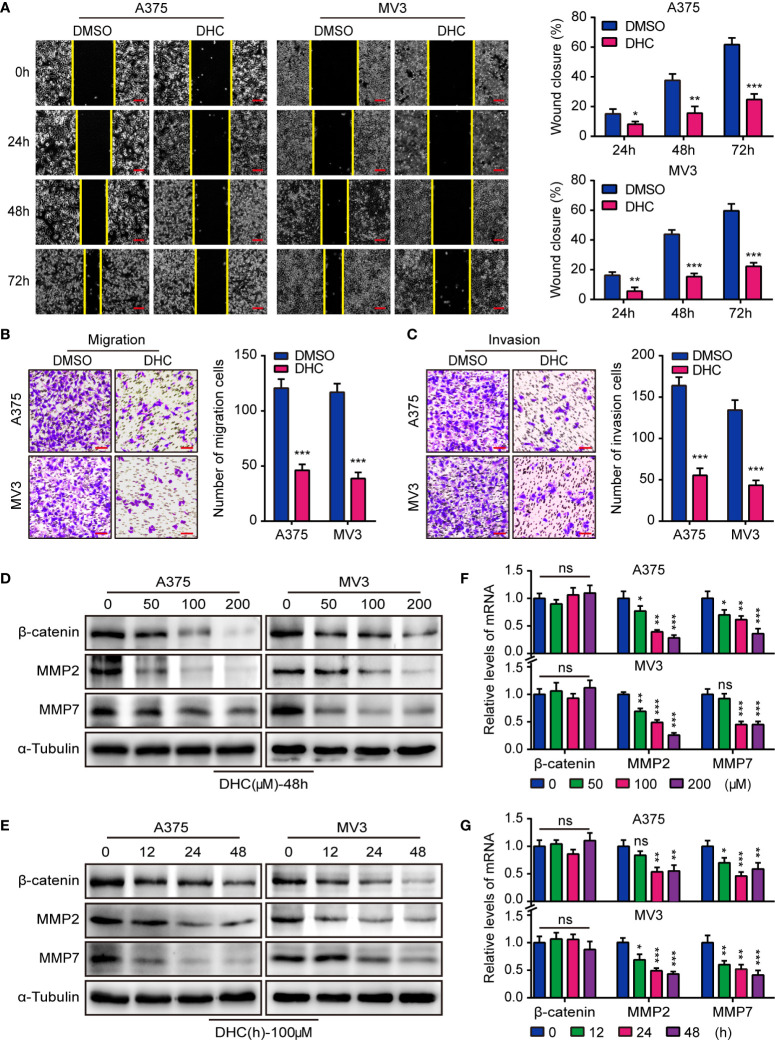
DHC inhibits the migration and invasion of melanoma cells. **(A)** Wound closure proportion of A375 and MV3 cells under the treatment of 100 μM DHC. Scale bar, 100 μm. **(B, C)** Migration and invasion transwell assays were performed in A375 and MV3 cells treated with 100 μM DHC. Scale bar, 100 μm. **(D, F)** The protein and mRNA levels of β-catenin, MMP2, and MMP7 in A375 and MV3 cells treated with 0, 50, 100, and 200 μM DHC for 48 h. **(E, G)** The protein and mRNA levels of β-catenin, MMP2, and MMP7 in A375 and MV3 cells treated with 100 μM DHC for 0, 12, 24, and 48 h. *P < 0.05; **P < 0.01; ***P < 0.001; ns, not significant.

In consideration of the high aggressiveness of melanoma, we investigated whether DHC had an impact on cell migration and invasion. Wound healing assays showed that DHC significantly retarded the wound closure rate (**Figure 2A**). Transwell assay results further revealed that the migration and invasion abilities of A375 and MV3 cells were repressed by DHC (**Figures 2B, C**). The expression of β-catenin, MMP2, and MMP7 proteins, which involved in cell migration and invasion, were remarkably down-regulated in a dose- and time-dependent manner in DHC treated cells (**Figures 2D, E**). The mRNA expression levels of MMP2 and MMP7 were also decreased in a dose- and time-dependent manner after DHC treatment, while there was no significant change in β-catenin (**Figures 2F, G**). Taken together, these results indicated that DHC remarkably suppressed the migration and invasion of melanoma cells.

### DHC Inhibits Tumor Genesis, Growth, and Metastasis of Melanoma Cells

**Figure 3:**
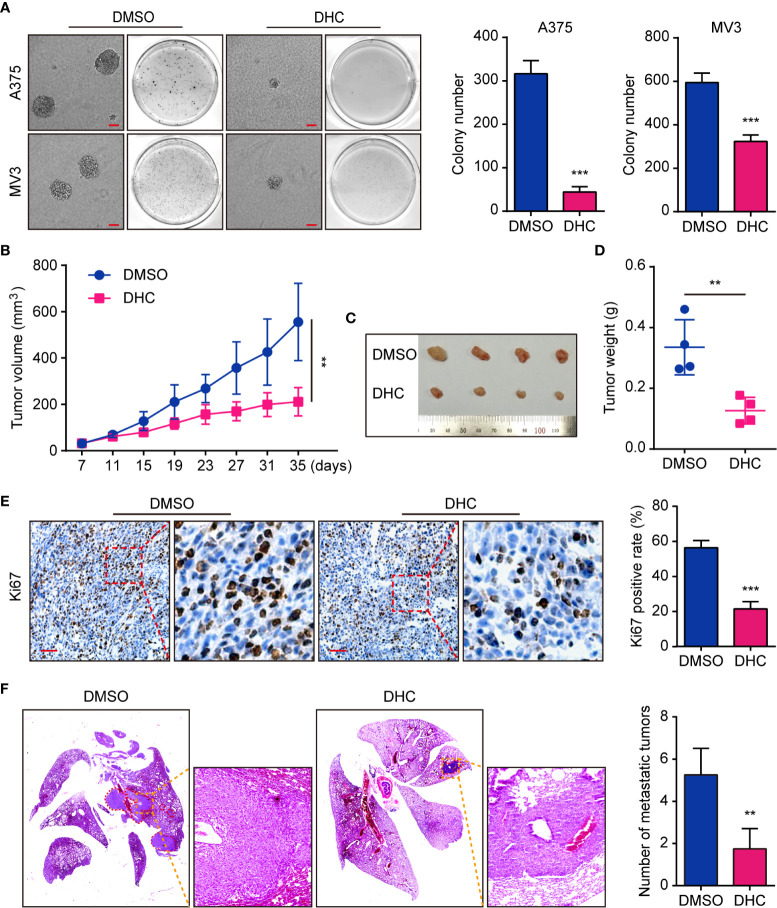
DHC suppresses tumorigenesis and pulmonary metastasis of melanoma cells. **(A)** Colonies generated by A375 and MV3 cells after treatment with 100 μM DHC for 3 weeks. Scale bar, 1 mm. **(B)** Tumor volume of A375 xenograft tumors in mice treated with DHC (20 mg/kg/day for 28 days) and DMSO. **(C, D)** The tumors in mice were excised and weighed. **(E)** IHC of Ki67 in the xenograft tumors. Scale bar, 100 μm. **(F)** H&E staining of the lungs from A375 metastasis mice model after treatment with DHC (20 mg/kg/day for 45 days) and DMSO. **P < 0.01; ***P < 0.001.

On account of the consequence that DHC inhibited melanoma cells proliferation, we tested the effect of DHC on colony formation *in vitro*. Soft agar assay showed that the DHC-treated A375 and MV3 cells generated smaller and fewer colonies compared with DMSO groups (**Figure 3A**). In order to explore whether it has the same effect *in vivo*, we generated an A375 xenograft tumor model in NOD/SCID mice. Compared with the control group, DHC significantly inhibited tumor growth (**Figures 3B–D**), and markedly decreased the expression of cell proliferation marker Ki67 in the tumor tissues (**Figure 3E**). Furthermore, the pulmonary metastasis of A375 cells was blocked by DHC in tumor metastasis mice model (**Figure 3F**). All these findings suggest that DHC effectively inhibits tumor growth and metastasis of melanoma cells *in vivo*.

### Overexpression of β-catenin Rescued DHC-Induced Inhibition of Proliferation in Melanoma Cells

**Figure 4:**
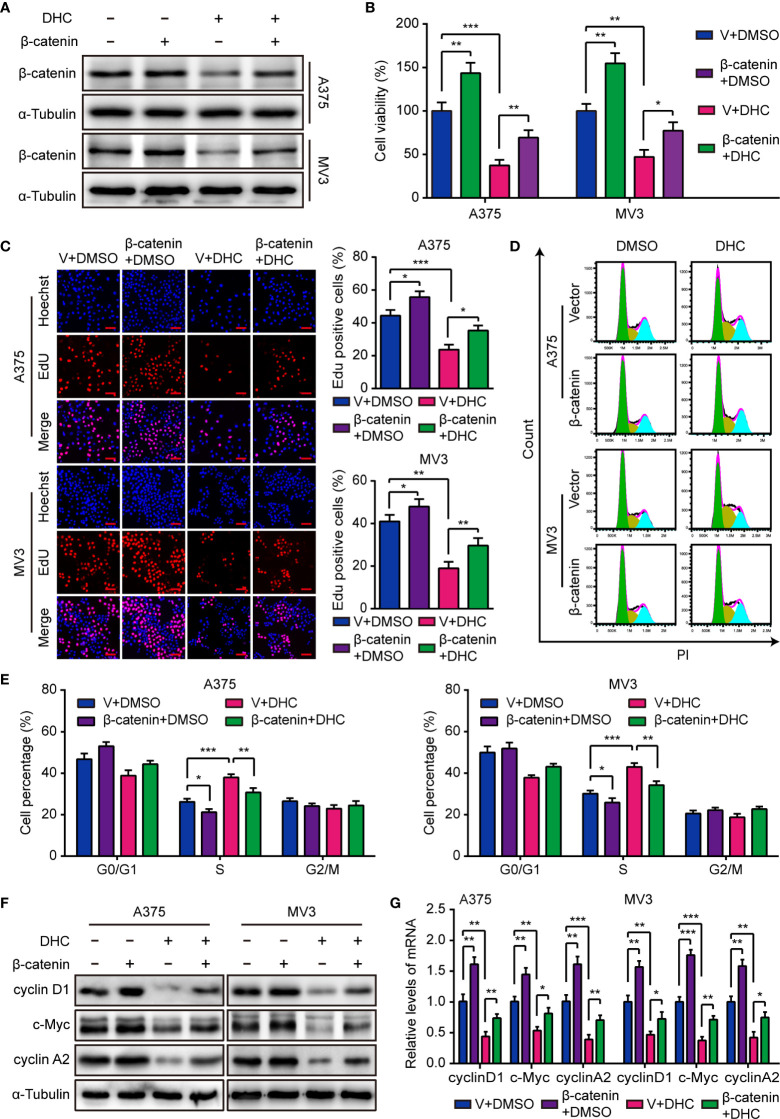
Overexpression of β-catenin retrieves DHC-induced cell proliferation inhibition. **(A)** The protein expression of β-catenin in β-catenin/vector overexpressed A375 and MV3 cells treated with 100 μM DHC for 48 h. **(B)** Cell viabilities of β-catenin/vector overexpressed A375 and MV3 cells treated with 100 μM DHC for 48 h. **(C)** EdU staining of β-catenin/vector overexpressed A375 and MV3 cells treated with 100 μM DHC for 48 h. **(D, E)** Cell cycle analysis of β-catenin/vector overexpressed A375 and MV3 cells treated with 100 μM DHC for 48 h. **(F, G)** The protein and mRNA levels of cyclin D1, c-Myc, and cyclin A2 in β-catenin/vector overexpressed A375 and MV3 cells treated with 100 μM DHC for 48 h. *P < 0.05; **P < 0.01; ***P < 0.001.

As mentioned above, the protein level of β-catenin was markedly decreased after DHC treatment, as well as cyclin D1, c-Myc, MMP2, and MMP7. Considering that β-catenin can acts as a transcriptional regulator upon the genes of cyclin D1, c-Myc, MMP2, and MMP7 (34–37), we hypothesized that DHC might exert anti-cancer activity *via* a β-catenin dependent way. In order to testify this hypothesis, A375 and MV3 cells were forced to overexpress β-catenin (**Figure 4A**), and then the MTT, EdU, and flow cytometry assays were performed to test the effect of β-catenin overexpression. As data shown, the cell viability inhibition caused by DHC was effectively mitigated by β-catenin overexpression (**Figure 4B**). In addition, DHC-induced both EdU positive rate reduction and S phase arrest in melanoma cells were attenuated by elevated expression of β-catenin (**Figures 4C–E**). And the DHC-induced decrease in expression of cyclin D1, c-Myc, and cyclin A2, at both protein and mRNA levels, were partially rescued by β-catenin overexpression (**Figures 4F, G**). Taken together, it suggested that DHC inhibits melanoma cell proliferation in a β-catenin dependent manner.

### Overexpression of β-catenin Rescued DHC-Induced Migration and Invasion Suppression in Melanoma Cells

**Figure 5:**
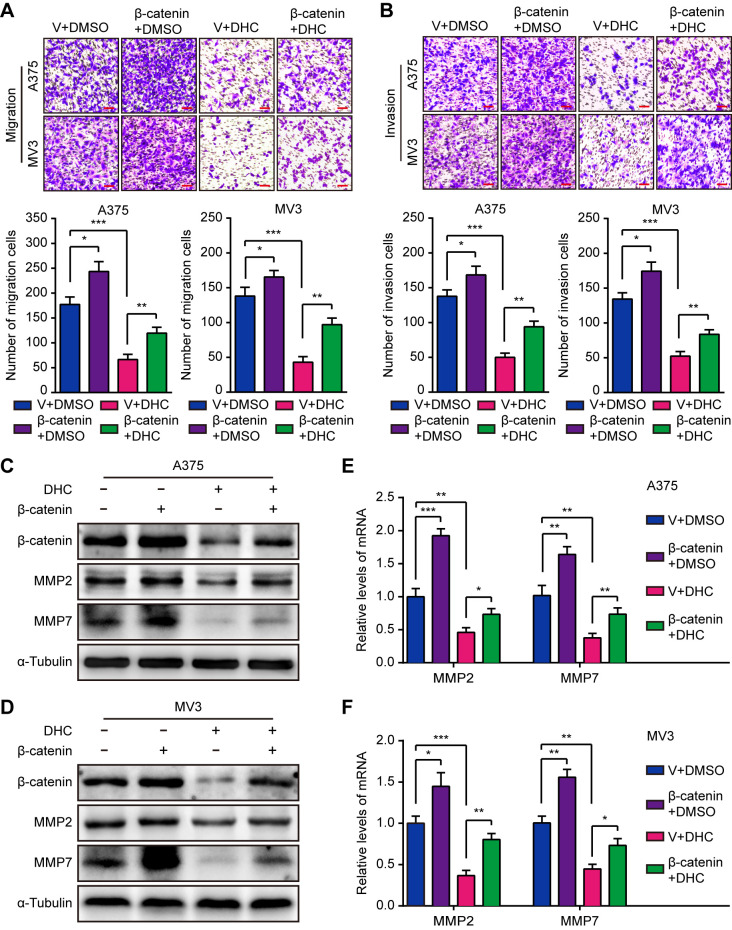
Overexpression of β-catenin retrieves DHC-induced cell migration and invasion inhibition. **(A, B)** Migration and invasion transwell assays were performed in β-catenin/vector overexpressed A375 and MV3 cells treated with 100 μM DHC. Scale bar, 100 μm. **(C, D)** The protein expression of β-catenin, MMP2, and MMP7 in β-catenin/vector overexpressed A375 and MV3 cells treated with 100 μM DHC for 48 h. **(E, F)** The mRNA expression of MMP2 and MMP7 in β-catenin/vector overexpressed A375 and MV3 cells treated with 100 μM DHC for 48 h. *P < 0.05; **P < 0.01; ***P < 0.001.

Since it has been confirmed that DHC inhibits melanoma cell proliferation through β-catenin suppression, we next explored whether it inhibited cell metastasis in the same manner. As expected, the DHC-induced inhibition of migration and invasion was partially rescued by elevated expression of β-catenin (**Figures 5A, B**). Congruently, the decreased MMP2 and MMP7 expression triggered by DHC was partially rescued by β-catenin overexpression, at both protein and mRNA levels (**Figures 5C–F**). These results indicated that β-catenin is a key factor of the DHC-induced suppression of melanoma cells migration and invasion.

### Overexpression of β-catenin Retrieves DHC-Induced Tumor Growth and Metastasis Inhibition

**Figure 6:**
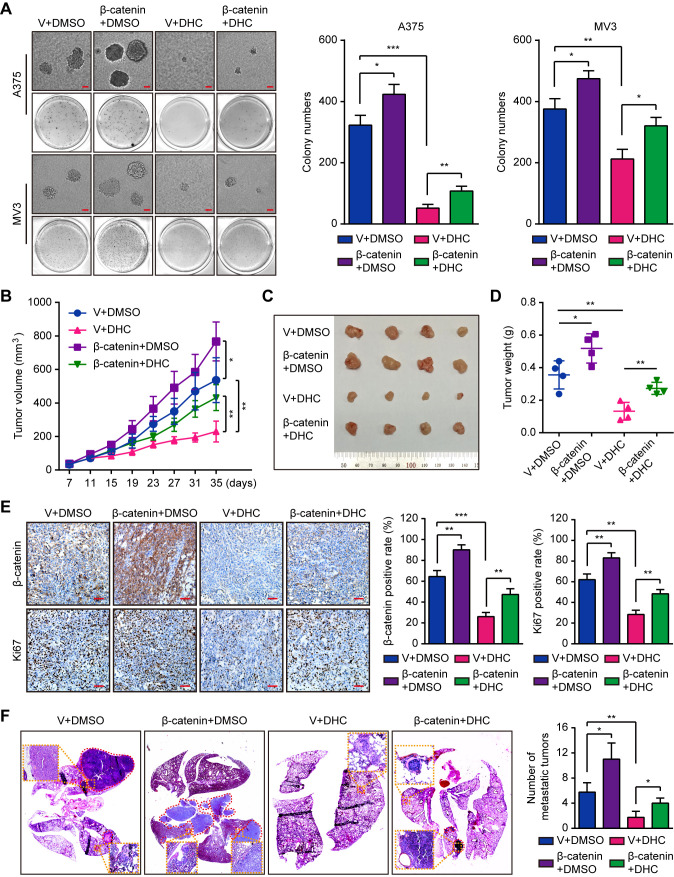
Overexpression of β-catenin retrieves DHC-induced inhibition of tumor growth and pulmonary metastasis of melanoma cells. **(A)** Colonies generated by β-catenin/vector overexpressed A375 and MV3 cells after treatment with 100 μM DHC for 3 weeks. Scale bar, 1 mm. **(B)** Tumor volume of β-catenin/vector overexpressed A375 xenograft tumors in mice after treatment with DHC (20 mg/kg/day for 28 days) and DMSO. **(C, D)** The tumors in mice were excised and weighed. **(E)** IHC of β-catenin and Ki67 in the xenograft tumors. Scale bar, 100 μm. **(F)** H&E staining of the lungs from β-catenin/vector overexpressed A375 metastasis mice model after treatment with DHC (20 mg/kg/day for 45 days) and DMSO. *P < 0.05; **P < 0.01; ***P < 0.001.

Subsequently, we explored the impact of β-catenin on tumorigenesis. Results of soft agar assays showed that β-catenin overexpression partly rescued the DHC-induced inhibition on colony formation *in vitro* (**Figure 6A**). *In vivo*, overexpression of β-catenin notably reduced the effect of DHC on tumor growth inhibition (**Figures 6B–D**), which was also shown in the change of Ki67 in tumor tissues (**Figure 6E**). Furthermore, the DHC-induced blockage on pulmonary metastasis of melanoma cells in mice model was partly removed by β-catenin overexpression (**Figure 6F**). All these findings suggested that DHC inhibited tumor growth and metastasis of melanoma cells in a β-catenin dependent manner.

### DHC Regulates β-catenin Expression Through a Ubiquitination Degradation Pathway

**Figure 7:**
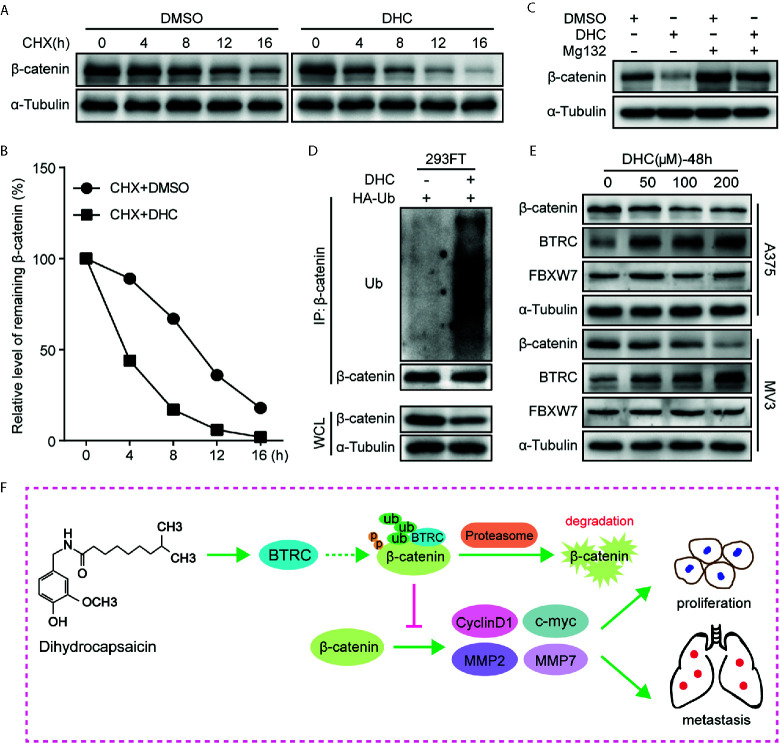
DHC down-regulates β-catenin by increasing its ubiquitination. **(A, B)** The protein turnover rate of β-catenin in A375 cells under the treatment of 100 μM DHC. **(C)** The protein expression of β-catenin in 100 μM DHC or DMSO treated cells in the presents or absents of MG132. **(D)** Ubiquitination levels of β-catenin was detected in HA-Ub transfected 293FT cells after treatment with DHC. **(E)** The expression of BTRC and FBXW7 in A375 and MV3 cells treated with 0, 50, 100, and 200 μM of DHC for 48 h. **(F)** Model of the impact of DHC on melanoma cells by the regulation of β-catenin signaling pathway was shown.

As β-catenin is the key effector, the underlying mechanisms by which DHC regulates β-catenin was further determined. Unlike the protein expression, the mRNA level of β-catenin was barely changed after DHC treatment (**Figures 2F, G**), which led us to consider the ubiquitination degradation pathway. As expected, DHC significantly accelerated the degradation of β-catenin protein (**Figures 7A, B**), while the degradation was blocked by 20 μM MG132 (**Figure 7C**). The ubiquitination assay confirmed that DHC significantly increased the ubiquitination level of β-catenin (**Figure 7D**). Subsequently, the expression of BTRC and FBXW7, which are E3 ubiquitin ligases of β-catenin, was examined by western blotting. The results showed that FBXW7 was unchanged while BTRC was dramatically up-regulated after DHC treatment (**Figure 7E**). The docking energies of DHC with β-catenin and BTRC as simulated by iGEMDOCK were −82.7 and −72.5 kcal/mol respectively, which indicates a good binding affinity (**Figure S2**). In summary, DHC could enhance the expression of BTRC and may also promote β-catenin and BTRC binding, thus accelerating the ubiquitination degradation of β-catenin. Working model for anti-cancer effect of DHC through regulating β-catenin pathway in melanoma was proposed (**Figure 7F**).

## Discussion

Malignant melanoma has long been the most lethal cutaneous tumor for its strong metastatic power and the shortage of effective therapeutic strategies. Despite recent advances such as the application of targeted agents and immune-checkpoint inhibitors in therapy of advanced melanoma, it is still urgent to find novel therapy strategies for malignant melanoma due to the therapeutic resistance, side-effects, and relapse (19, 38). DHC is a plant-derived organic component from Capsicum with multiple pharmacological effects, including anti-inflammation, anti-microbial, and anti-oxidant (39–41). Although previous studies have shown that DHC exhibited anticancer activity in some tumors, its effect against melanoma has not been reported.

In this study, DHC effectively inhibited the proliferation, tumor genesis, migration, and invasion of melanoma cells *in vitro*. Moreover, the tumor growth and pulmonary metastasis of melanoma cells were significantly suppressed by DHC *in vivo*. According to our data, the protein levels of β-catenin and its downstream factors cyclin D1, c-Myc, MMP2, and MMP7 were all markedly decreased in melanoma cells after DHC treatment. Therefore, it is hypothesized that DHC exerted its inhibitory effect in a β-catenin dependent pattern. Thus, exogenous β-catenin was forced to overexpress in melanoma cells, which significantly attenuated the suppression effects of DHC on melanoma cells, both *in vitro* and *in vivo*.

β-catenin is a multifunctional protein which acts as a pivotal signal transducer of the Wnt/β-catenin pathway. In the absence of Wnt ligands, free cytoplasmic β-catenin is recruited and phosphorylated by a destruction complex formed by axis inhibitor (AXIN), adenomatous polyposis coli (APC), casein kinase 1 (CK1), and glycogen synthase kianase-3β (GSK3β), then the phosphorylated β-catenin is degraded by the ubiquitin-proteasome system (42, 43). Once Wnt is activated by its ligands, the cytoplasmic phosphorylation of β-catenin and its degradation are inhibited. Consequently, the unphosphorylated β-catenin accumulates in the cytoplasm and translocates into the nucleus where it forms active transcriptional complexes with LEF/TCF to promote the transcription of target genes, including cyclin D1, c-Myc, MMP2, and MMP7 (34–37).

C-Myc is a well-recognized oncogene, which is involved in various processes like cell proliferation, anti-apoptosis, and metastasis during tumorigenesis progression (44). It is reported that c-Myc overexpression drives melanoma metastasis by promoting vasculogenic mimicry *via* c-Myc/snail/Bax signaling (45). In addition, c-Myc can accelerate S-phase progress in the cell division cycle (46). Cyclin D1, known as an essential cell cycle regulator, has also been proved participated in tumor cellular migration and invasion (47). MMP2 and MMP7 belong to the family of zinc-dependent endopeptidases, which play a fundamental role in tumor invasion and metastasis. They degrade extracellular matrix and allow the tumor cells to invade into the surrounding tissues and migrate to other organs (48, 49). Clinical studies showed significantly increased expression of MMP2 and MMP7 in melanomas, while patients with strong MMP2 or MMP7 had a bad survival (50, 51).

Ubiquitin-proteasome mediated degradation plays a key role in the regulation of β-catenin. BTRC is an E3 ubiquitin ligase well studied in ubiquitination of β-catenin (52). BTRC interacts with S-phase-associated kinase 1 (SKP1) and Cullin1 to form SCF (SKP1-Cullin1-F-box protein) E3 ubiquitin ligase complex, thus to ubiquitinate β-catenin (53). In this research, the protein ubiquitination of β-catenin after DHC treatment was explored and the results indicated that DHC might promote the ubiquitination degradation of β-catenin through upregulating BTRC. In addition to this, according to molecular docking data, DHC may also recruit β-catenin to combine with BTRC. However, it remains unclear how DHC regulates the expression of BTRC, which needs further exploration. Ubiquitination and degradation will reduce the accumulation of β-catenin in cytoplasm and then the translocation of β-catenin to the nucleus decreases accordingly. This is reflected by the decreased nuclear β-catenin staining in IHC of DHC-treated xenograft tumors (as shown in **Figure 6E**), although the positive rate has not been calculated in detail. On the other hand, the expression of those genes transcriptionally regulated by β-catenin, which were mentioned above, was reduced both in mRNA and protein level after DHC treatment and can be rescued by overexpression of β-catenin, further illustrated this situation.

In conclusion, our present study shows that DHC can significantly suppress the proliferation and metastasis of melanoma cells through the inhibition of β-catenin pathway. Thus, DHC could be considered as a potential agent for human melanoma therapy, while β-catenin may be one of the therapeutic targets.

## Data Availability Statement

The raw data supporting the conclusions of this article will be made available by the authors, without undue reservation.

## Ethics Statement

The animal study was reviewed and approved by the Committee for Animal Protection and Utilization of Southwest University.

## Author Contributions

SS and CL performed most of the experiments and wrote the original manuscript. YZ, CD, WL, and JD performed part of the experiments. YJ and LG provided reagents and technical assistance. LL and HH conducted the data collection and statistical analysis. HC and QL provided fund support. YL and HC designed and supervised the project, and modified the manuscript. All authors contributed to the article and approved the submitted version.

## Funding

This work was supported by the National Natural Science Foundation of China (81872071 and 81672502); the Natural Science Foundation of Chongqing (cstc2019jcyj-zdxmX0033); Fundamental Research Funds for the Central Universities (XYDS201912); and the Major Medical Research Projects in Hebei Province (20180412).

## Conflict of Interest

The authors declare that the research was conducted in the absence of any commercial or financial relationships that could be construed as a potential conflict of interest.
