# Corrigendum: Dihydrocapsaicin inhibits cell proliferation and metastasis in melanoma *via* down-regulating β-catenin pathway

**DOI:** 10.3389/fonc.2022.1052365

**Published:** 2022-10-11

**Authors:** Shaomin Shi, Chongyang Li, Yanli Zhang, Chaowei Deng, Wei Liu, Juan Du, Qian Li, Yacong Ji, Leiyang Guo, Lichao Liu, Huanrong Hu, Yaling Liu, Hongjuan Cui

**Affiliations:** ^1^ Department of Dermatology, Third Hospital of Hebei Medical University, Shijiazhuang, China; ^2^ State Key Laboratory of Silkworm Genome Biology, Southwest University, Chongqing, China; ^3^ Cancer Center, Medical Research Institute, Southwest University, Chongqing, China; ^4^ Department of Dermatology, Fifth Hospital of Shijiazhuang, Shijiazhuang, China

**Keywords:** dihydrocapsaicin, melanoma, cell proliferation, metastasis, β-catenin, ubiquitination

In the original article, there were mistakes in **Figures 5** and **6** as published. We found that the migration transwell assay image of “A375-Vector+DMSO” in **Figure 5A**, invasion transwell assay images of “A375-Vector+DHC”, “MV3-β-catenin+DHC” in **Figure 5B**, and soft agar assay image of “A375-Vector+DHC” in **Figure 6A** were inadvertently presented with incorrect pictures. The corrected **Figures 5** and its caption “Overexpression of β-catenin retrieves DHC-induced cell migration and invasion inhibition. **(A, B)** Migration and invasion transwell assays were performed in β-catenin/vector overexpressed A375 and MV3 cells treated with 100 μM DHC. Scale bar, 100 μm. **(C, D)** The protein expression of β-catenin, MMP2 and MMP7 in β-catenin/vector overexpressed A375 and MV3 cells treated with 100 μM DHC for 48 h. **(E, F)** The mRNA expression of MMP2 and MMP7 in β-catenin/vector overexpressed A375 and MV3 cells treated with 100 μM DHC for 48 h. *P < 0.05; **P < 0.01; ***P < 0.001” and **Figure 6** and its caption **“**Overexpression of β-catenin retrieves DHC-induced inhibition of tumor growth and pulmonary metastasis of melanoma cells. **(A)** Colonies generated by β-catenin/vector overexpressed A375 and MV3 cells after treatment with 100 μM DHC for 3 weeks. Scale bar, 1 mm. **(B)** Tumor volume of β-catenin/vector overexpressed A375 xenograft tumors in mice after treatment with DHC (20 mg/kg/day for 28 days) and DMSO. **(C, D)** The tumors in mice were excised and weighed. **(E)** IHC of β-catenin and Ki67 in the xenograft tumors. Scale bar, 100 μm. **(F)** H&E staining of the lungs from β-catenin/vector overexpressed A375 metastasis mice model after treatment with DHC (20 mg/kg/day for 45 days) and DMSO. *P < 0.05; **P < 0.01; ***P < 0.001” appear below.

**Figure 5:**
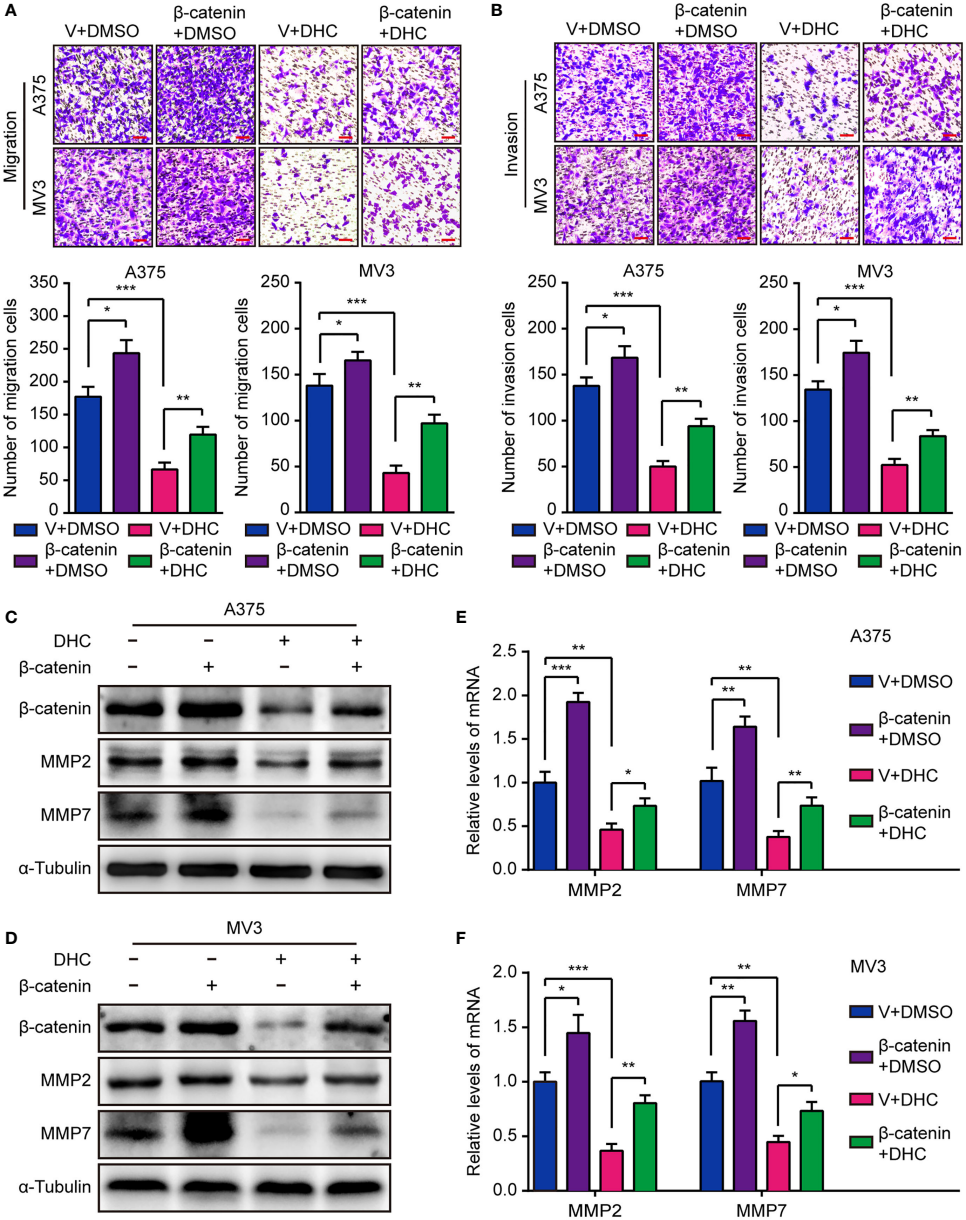
Overexpression of β-catenin retrieves DHC-induced cell migration and invasion inhibition. **(A, B)** Migration and invasion transwell assays were performed in β-catenin/vector overexpressed A375 and MV3 cells treated with 100 μM DHC. Scale bar, 100 μm. **(C, D)** The protein expression of β-catenin, MMP2 and MMP7 in β-catenin/vector overexpressed A375 and MV3 cells treated with 100 μM DHC for 48 h. **(E, F)** The mRNA expression of MMP2 and MMP7 in β-catenin/vector overexpressed A375 and MV3 cells treated with 100 μM DHC for 48 h. *P < 0.05; **P < 0.01; ***P < 0.001.

**Figure 6:**
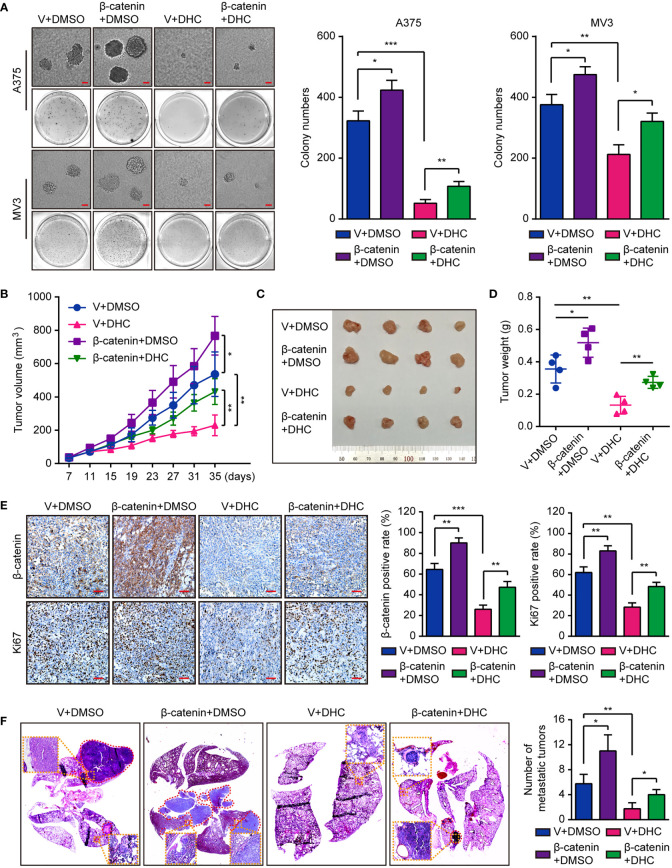
Overexpression of β-catenin retrieves DHC-induced inhibition of tumor growth and pulmonary metastasis of melanoma cells. **(A)** Colonies generated by β-catenin/vector overexpressed A375 and MV3 cells after treatment with 100 μM DHC for 3 weeks. Scale bar, 1 mm. **(B)** Tumor volume of β-catenin/vector overexpressed A375 xenograft tumors in mice after treatment with DHC (20 mg/kg/day for 28 days) and DMSO. **(C, D)** The tumors in mice were excised and weighed. **(E)** IHC of β-catenin and Ki67 in the xenograft tumors. Scale bar, 100 μm. **(F)** H&E staining of the lungs from β-catenin/vector overexpressed A375 metastasis mice model after treatment with DHC (20 mg/kg/day for 45 days) and DMSO. *P < 0.05; **P < 0.01; ***P < 0.001.

The authors apologize for these errors and state that this does not change the scientific conclusions of the article in any way. The original article has been updated.

## Publisher’s note

All claims expressed in this article are solely those of the authors and do not necessarily represent those of their affiliated organizations, or those of the publisher, the editors and the reviewers. Any product that may be evaluated in this article, or claim that may be made by its manufacturer, is not guaranteed or endorsed by the publisher.

