# Supplementary material for: Dihydrocapsaicin Inhibits Cell Proliferation and Metastasis in Melanoma via Down-regulating β-Catenin Pathway
Source: Front Oncol. 2021 Mar 23;11:648052. doi: 10.3389/fonc.2021.648052 (PMC8023049; doi:10.3389/fonc.2021.648052)
Supplement: Supplementary file 1 [file DataSheet_1.docx]

Supplementary Material

## Supplementary Figures


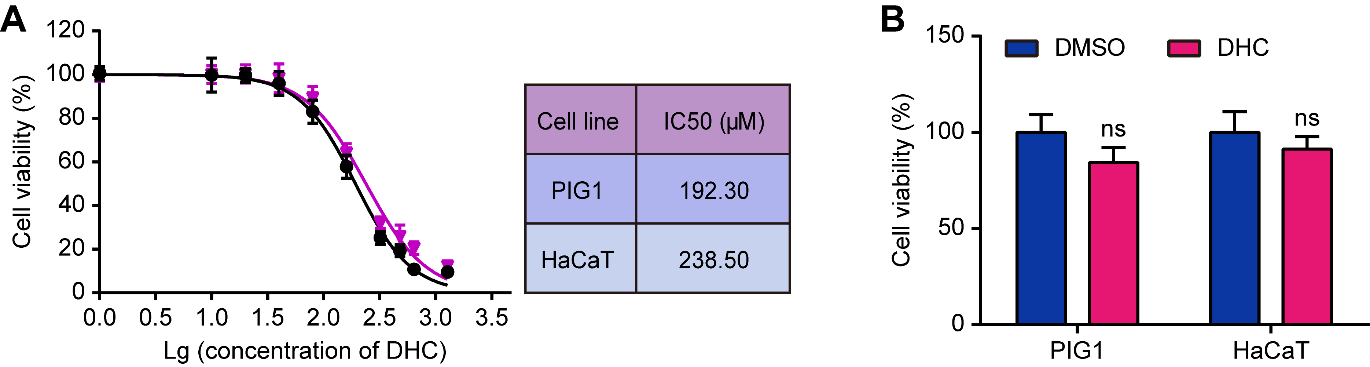


**Supplementary Figure 1.** The effects of DHC on PIG1 and HaCaT cells. **(A)** The IC50 of DHC for PIG1 and HaCaT cells were measured. **(B)** Cell viabilities of PIG1 and HaCaT cells treated with 100 μM of DHC for 48 h.


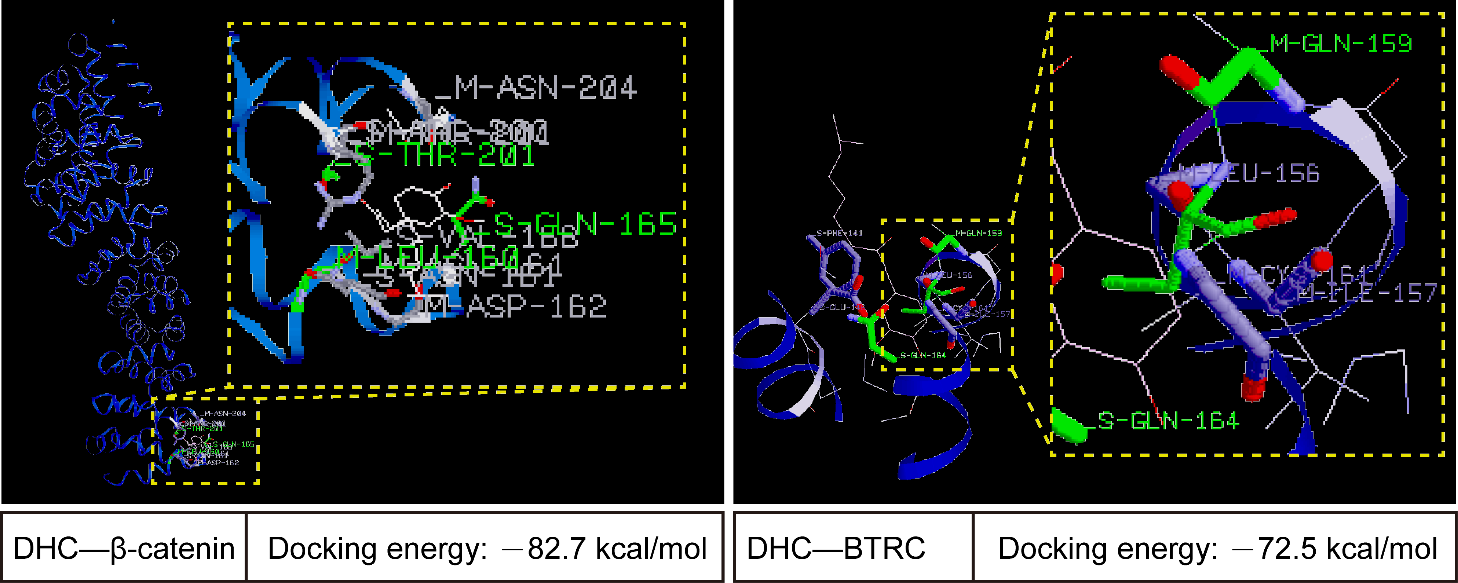


**Supplementary Figure 2.** Molecular docking of DHC with β-catenin and BTRC.
